# Editorial: A Genetic Perspective on Asian Populations

**DOI:** 10.3389/fgene.2022.883843

**Published:** 2022-06-08

**Authors:** Wibhu Kutanan, Piya Changmai, Chuan-Chao Wang

**Affiliations:** ^1^ Department of Biology, Faculty of Science, Khon Kaen University, Khon Kaen, Thailand; ^2^ Department of Biology and Ecology, Faculty of Science, University of Ostrava, Ostrava, Czechia; ^3^ Department of Anthropology and Ethnology, School of Sociology and Anthropology, Institute of Anthropology, Xiamen University, Xiamen, China; ^4^ State Key Laboratory of Cellular Stress Biology, School of Life Sciences, Xiamen University, Xiamen, China; ^5^ State Key Laboratory of Marine Environmental Science, Xiamen University, Xiamen, China

**Keywords:** genetic ancestry, genetic markers, asia, asian populations, genetic admixture

Asia is the world’s largest and most geographically and ethnolinguistically diverse continent. Those various ethnolinguistic groups inhabit different geography and climatic extremes. Such linguistic and geographic diversifications could be correlated with the genetic variation of human populations. So far, several previous studies have reported on the genetic variation of Asian populations. Since there is an enormous diversity of modern human populations in Asia, abundant present-day populations and numerous human remains have been left for investigation. Therefore, we established this Research Topic and we initially intended to call for research articles on populations from all regions across Asia. However, we were not able to recruit research papers on populations from some parts of the continent. Nevertheless, the papers in our Research Topic still cover diverse ethnolinguistic groups of vast regions of Asia. Finally, we collected one review article and a total of 13 research papers that examined genetic variations from diverse present-day Asian populations and ancient DNA data using different kinds of genetic markers and methods.

We summarized the main results of each study according to geographic location; we began with Northern East Asia/North of China. Wei investigated the association between bi-allelic polymorphisms of two genes: *ACE* (I/D) and *ACTN3* (R/X), and some anthropometric, physical and strength traits of rowing athletes who are ethnically Han Chinese from the north of China. The alleles of these genes were genotyped by conventional PCR-based method, and allelic frequencies of the studied groups were compared between genders and between populations, specifically the Han Chinese and East Asian groups. The results showed an association between *ACE* I allele and XX genotype and male endurance traits, while *ACE* D allele is associated with female strength traits. The author also compared and discovered some differences in the frequency of *ACTN3* R/X alleles between rowers and other Chinese populations, suggesting the application of using these polymorphisms as biomarkers of genetic traits in Chinese rowing athletes. Dong et al. reported allelic frequency of 23 single nucleotide polymorphisms (SNPs) on eight skeletal muscle strength-related genes in five northern Chinese ethnic groups from Heilongjiang Province: Hezhen, Manchu, Daur, Ewenki, and Mongolian. The latter three groups showed close genetic affinity. There are statistically genetic differences between rs1815739 (*ACTN3* gene) and rs7975232 (*VDR* gene) among the five ethnic groups, and the authors suggested that natural selection could explain these results. Another association study was conducted by Cho et al.; the authors investigated the correlation between genetic signals and nine anthropometric traits, e.g. height, weight and body mass index. They analysed genome-wide data generated by KoreanChip from a huge number of Korean participants. They successfully identified nine novel genetic variants: eight in chromosome 6 at the gene *RP11-513I15.6* and one in chromosome 12 near the gene *RP11-977G19.10* that are associated with height in Korean females. The authors also found six Asian-specific genetic variants that could be developed for further investigation on forensic anthropology.

Another genome-wide study using Illumina Array of Mongolians from Baotou city of Inner Mongolia Autonomous Region was done by (Yang et al.). Merged data from present-day East Asian populations and ancient samples across Asia were intensively analysed with multiple tests to explore Mongolian genetic architecture. In consistent with prehistorical and historical evidence that indicated complex demographic movements in Eastern Eurasia, the Mongolian showed three genetic components: one enriched in present-day Sino-Tibetan speaking groups/ancient Neolithic millet farmers of Yellow River Basin, one from ancient Neolithic hunter-gatherers of North Asia and another from Western Eurasian-related ancestral component related to the Western Steppe herders and Iranian farmers. The authors also revealed signals of recent positive selection in the *MHC* region that related to the human immune response. Zhou et al. developed methods for multiplex amplification and genotyping of 52 ancestry informative deletion/insertion polymorphisms (AIDIPs) and tested its efficiency with the Han population from Eastern China. The authors compared a subset of these AIDIPs with populations from African, European, American and South Asian and found genetic distinction of East Asian from other groups. The Eastern Han exhibited close genetic relatedness to East Asians and obtained East Asian ancestral components with high proportions. Because this AIDIPs panel had high cumulative discrimination power in Eastern Han, the authors suggested that this panel could be beneficial for additional forensic investigation apart from the commonly used method. Another study by Zhang et al. genotyped 38 X-chromosome InDel polymorphisms (X-InDels) of Han Chinese from Henan Province. This forensic investigation exhibited the effectiveness of this 38 X-InDels panel as a complementary tool for forensic applications, e.g. individual identification and parentage testing of trios, though this panel can only differentiate inter-continental populations but not intra-continental groups. The authors suggested further study could improve the power of this panel with other compound makers.

In Northwestern China, Wang et al. sequenced mitochondrial (mt) DNA in the control region using a massively parallel sequencing platform in the Altaic-speaking Kirgiz group from Northwest China. The authors compared sequence data of Kirgiz with reference populations worldwide. The Kirgiz had more maternal relatedness to East Asian populations; the mtDNA haplogroups of the Kirgiz group were ∼70% of East Asian prevalent haplogroups and ∼30% of haplogroups from West Asia.

The Maoming Han from Guangdong Province of Southeastern China were regarded as the descendants of Gaoliang aborigines. Maoming Han speak Cantonese, which is another branch of the Sino-Tibetan language family. Fan et al. studied the genetic structure of Maoming Han from Guangdong using forensic markers. Genotypes of 27 Y-STRs from 431 Maoming Han were done with AmpFLSTR^®^ Yfiler^®^ Plus PCR Amplification Kit. The authors reported allelic frequency and forensic parameters that support the effectiveness of using this marker set for forensic applications in Maoming Han. The Maoming Han exhibited closest paternal relatedness to Hakka, another Han Chinese group from Guangdong Province.

Southwestern East Asia/southwestern China is the region that harbors geographic, cultural and ethnolinguistic diversities. The Tai-Kadai and Hmong-Mien speaking populations were groups distributed in this region. Chen et al. employed the battery of Infinium Global Screening Array to generate genome-wide data of Tai-Kadai speaking-Maonan from Guizhou Province. The authors included previously published present-day and ancient East Asian and Southeast Asian populations for genetic comparison. The fine-scale genetic structure of Maonan was explored; the Guizhou Maonan had the closest genetic relationship with Guangxi Maonan. Both of them showed genetic affinity to other geographically Tai-Kadai speaking populations. Genetic admixtures with neighboring Guizhou populations were also observed. The ancestries of Tai-Kadai speaking populations were mainly related to ancient southern East Asians with an additional minor proportion from northern East Asians. Bin et al. study the fine-scale genetic structure of Tai-Kadai speaking Sui from Guangxi and Guizhou using genome-wide SNPs; there are substructures of Guizhou Sui and Guangxi Sui. The former showed relationships with other Tai-Kadai speaking groups in the vicinity, while the latter had additional genetic components from Hmong-Mien-speaking populations and Northern East Asians. In general, the Sui populations showed close genetic relatedness to various southern Chinese and Southeast Asian populations, particularly Tai-Kadai and Hmong-Mien speaking groups. The Sui also showed a genetic affinity with ancient individuals from southern China, supporting southern China as their original place.

Using the same platform as Chen et al., Liu et al. analysed genome-wide data of the Sichuan Miao group together with other Hmong-Mien speaking groups from China and Southeast Asia and also with other present-day and ancient data across East Asia. There is a new ancestral lineage that existed in the Hmong-Mien speaking groups suggesting their common origin. However, some interactions in different areas also influenced the genetic structure of some Hmong-Mien speaking groups, e.g. Dao from Vietnam, Iu Mien from Thailand and Miao and She from Chongqin. With analyses of sharing patterns of the new ancestral lineage specific to the Hmong-Mien groups and estimated admixture times, the authors suggested that Southwest China was the original place of Hmong-Mien speaking groups then recently migrated southward to Mainland Southeast Asia. Apart from the Tai-Kadai, Hmong-Mien, and Sino-Tibetan speaking groups which scattered in Southwestern China, the Mongolic-speaking Mongolians and Tungusic-speaking Manchus were also inhabited in this area. The Mongolian and Manchus moved from the North to Guizhou not older than 800 years ago. Chen et al. compared Guizhou Mongolian and Manchus with multiple present-day and ancient East Asian populations. The southwestern Mongolic and Tungusic speaking groups had mixed ancestries: one related to northern ancestor and one related to southern indigenous East Asian. The admixture dating results are consistent with historical evidence that indicated admixture events during the Mongolians Empire expansion during the formation of the Yuan dynasty.

Southwestern China shares a border with the Tibetan Plateau to the west. With extreme environment for human occupation, e.g. high altitude, perennial low temperature, extreme aridity and severe hypoxia, geneticists have paid much attention to this area to study genetic adaptation. He et al. recruited genome-wide data generated from different platforms from previous studies and investigated the fine genetic structure of the Tibetan groups from geographically diverse regions: Ü-Tsang Tibetan, Ando Tibetan and Kham Tibetan groups compared with present-day and ancient samples. The authors found different genetic structures of three Tibetan groups which were possibly influenced by cultural and geographic factors. The Ü-Tsang Tibetans possessed a stronger Onge/Hoabinhian related affinity, Ando Tibetans harbored greater Western Eurasian related ancestry, and Kham Tibetans had much more Neolithic Southeast Asian ancestry.

There is only one article on Southeast Asia that submitted to this Research Topic. Hoh et al. reviewed the genetic studies of native groups in the Malay Peninsular and Borneo over the past century. The authors presented the contents according to chronology and types of biological and genetic markers that were generated data from primary to advance technologies: anthropological and physical traits, blood groups, protein variations, mitochondrial and autosomal DNA polymorphisms and whole-genome sequences.

In sum, albeit we were not able to recruit papers on populations from some parts of Asia, the papers in our Research Topic still cover populations of vast regions of Asia, especially East and Southeast Asia. The collection of fourteen articles in this Research Topic showed new population genetic results that were produced by heterogeneous technologies, reflecting the richness of research approaches in East/Southeast Asian genetics. Although nowadays many developments of advanced sequencing and microarray technologies and together with the innovative tools of bioinformatics have enabled researchers to deeply reconstruct population history based on modern and ancient DNA samples. We are still encouraging researchers who focused on STRs and InDels to provide a basic view of population structure, with overlapping advantages in forensic investigation. Further explorations with more present-day populations and ancient samples, particularly in Southeast Asia, will be provided new insights into the population structure, demographic history and natural selection of functional genes that could be useful for biomedical studies and forensic investigation in Asian populations.

